# The Utilization of Multiple-Walled Carbon Nanotubes in Polymer Modified Bitumen

**DOI:** 10.3390/ma10040416

**Published:** 2017-04-15

**Authors:** Benan Shu, Shaopeng Wu, Ling Pang, Barugahare Javilla

**Affiliations:** State Key Laboratory of Silicate Materials for Architectures, Wuhan University of Technology, Wuhan 430070, China; Shuba@whut.edu.cn (B.S.); wusp@whut.edu.cn (S.W.); makorogo@whut.edu.cn (B.J.)

**Keywords:** MWCNTs, SBS modified bitumen, rheological properties, modification mechanism

## Abstract

SBS (styrene-butadiene-styrene block copolymer) modified bitumen is one of most widely used polymer modified bitumens in China. It is also not satisfactory when subjected to extreme conditions. Multiple-walled carbon nanotubes, as a type of advanced nanomaterial, are investigated extensively because of their strong adsorption capacity. Little research has been done about MWCNTs/SBS modified bitumen, and in view of this, the performance and modification mechanism of MWCNTs/SBS modified bitumen was investigated in this paper. Conventional bitumen tests, Brookfield viscosity, bending beam rheometer, and dynamic shear rheometer tests showed improved performance at high and low temperature. The optimum MWCNTs content was determined as 1.0%. FT-IR, bitumen four components, and thermal analysis tests were conducted and revealed that the addition of MWCNTs led to a decrease in the content of light components. In addition, the rate of decomposition and volatilization of saturates and aromatics was reduced and better thermal stability of bitumen was found. Fluorescence microscopy tests showed that MWCNTs improved the dispersion of SBS and storage stability of the binder. Finally a schematic was proposed to explain how MWCNTs improved the performance of SBS modified bitumen through their strong adsorption property created by π–π intermolecular forces.

## 1. Introduction

The total mileage of bitumen highways in China reached 117,000 kilometers in 2016, and it is expected that there will be a rapid increase in the next few years. Highways are expected to have high temperature rutting resistance and low temperature crack resistance, and in view of this, modified bitumen such as SBS modified bitumen has shown a better rutting and cracking resistance compared to unmodified bitumen. The main problems identified with modifiers such as SBS have been its poor dispersion and compatibility with bitumen, and this is known to affect the rheological properties of bitumen [1].

In recent years, nanomaterials have been widely used to improve the rheological properties of bitumen, and to that effect, researchers have doped different nanoparticles [2,3]. Two specific types of nanomaterials, namely nano-metal oxides and nano-inorganic materials, have been widely investigated. Nano-metal oxides including nano-TiO_2_, nano-SiO_2_, and nano-ZnO could improve the rutting resistance of bitumen, but had little effect on its low temperature cracking resistance [4,5]. Nano-inorganic materials including nano-clay and nano-CNTs exhibited some special properties in bitumen because of their specific properties and structures. For example, nano-clay had a positive interaction effect with bitumen and polymer, because of the high compatibility between the clay and polymer which led to a better dispersion of the polymer in the bitumen. Nano-OMMT improved the thermal oxidative aging resistance of bitumen because of its specific layered structure which effectively blocked oxygen penetration in bitumen and thus improved thermal oxidative aging [6].

Carbon nanotubes (CNTs) are seamless nanotube structures containing monolayer or multilayer graphite sheets revolving at specific helix angles around the same axis. Each layer wall is a cylindrical surface composed of a hexagonal network plane made of sp2 hybrid carbon atoms [7]. Because of the overlap of P orbitals, the highly disjointed π-bond could exist at the outer layer of the CNTs. The non-covalent interaction between multi-wall carbon nanotubes (MWCNTs) and other macromolecules with conjugated properties such as aromatics and styrene exist because of the highly disjointed π-bond [8,9,10].

Amin et al. [11] used multi-wall carbon nanotubes (MWCNTs) as an additive to bitumen and it enhanced the high temperature performance of bitumen. Yang et al. [12] reported that single-wall carbon nanotubes (SWCNTs) as an additive to bitumen improved its penetration, softening point, ductility, and flash point properties. In bitumen mixtures, an optimum percentage of 0.05% of SWCNTs improved the Marshall stability and rigidity flow. Faramarzi [13] also investigated the effect of MWCNTs in bitumen and reported an increase of rutting factor$\;G^{*}/\sin\delta$. The study of Santagata et al. [14] showed that susceptibility to oxidative aging of bitumen was reduced with the addition of carbon nanotubes. Aging was expected to further improve with the long-term performance of bituminous mixtures.

SBS is widely used to improve the properties of bitumen such as rutting resistance, but it has been reported that the polystyrene in SBS was incompatible with bitumen. The attraction between polystyrene molecules resulted in the formation of insoluble agglomerates, and thus the swelling potential of SBS was limited [15]. Due to the highly disjointed π-bond of MWCNTs, a π–π interaction with polystyrene of SBS was expected, which would improve its compatibility with bitumen, and thus result in an improved rheological performance. For that reason, this paper conducted a series of experiments to determine the effect and mechanism of MWCNTs on SBS modified bitumen.

## 2. Experiments

### 2.1. Materials

**Figure 1:**
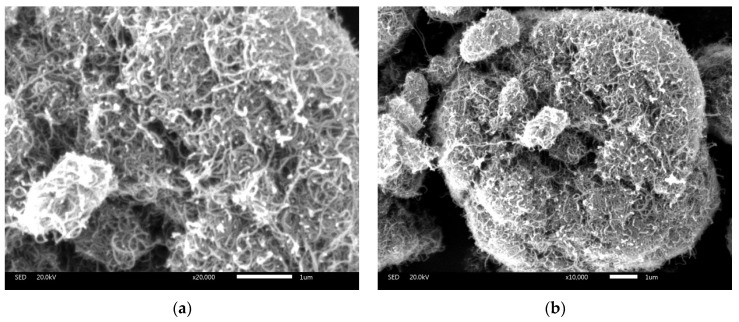
SEM images of MWCNTs (1 μm scale bar) at different magnification. (**a**) ×20,000; (**b**) ×10,000.

90A bitumen was supplied by KOCH Bitumen Co., Ltd. (Wuhan, China). SBS was bought from Dongguan Huahong Engineering Plastic Co., Ltd. (Dongguan, China). The type of SBS is Linear/161B. MWCNTs were bought from Suzhou Hengqiu Graphene Technology Co., Ltd. (Suzhou, China). The properties and micro-morphology of the MWCNTs are shown in Table 1 and Figure 1, respectively. From the SEM images it is seen that MWCNTs intertwined with each other because of their extremely large surface area, and so it was essential to use a high-speed shearing mixer to make them disaggregate in bitumen during the preparation of the MWCNTs/SBS modified bitumen. In order to determine the specific surface area of MWCNTs, the Specific surface area test (BET test) was conducted and the result was 213.6872 m^2^/g.

### 2.2. Preparation of MWCNTs/SBS Modified Bitumen

**Figure 2:**
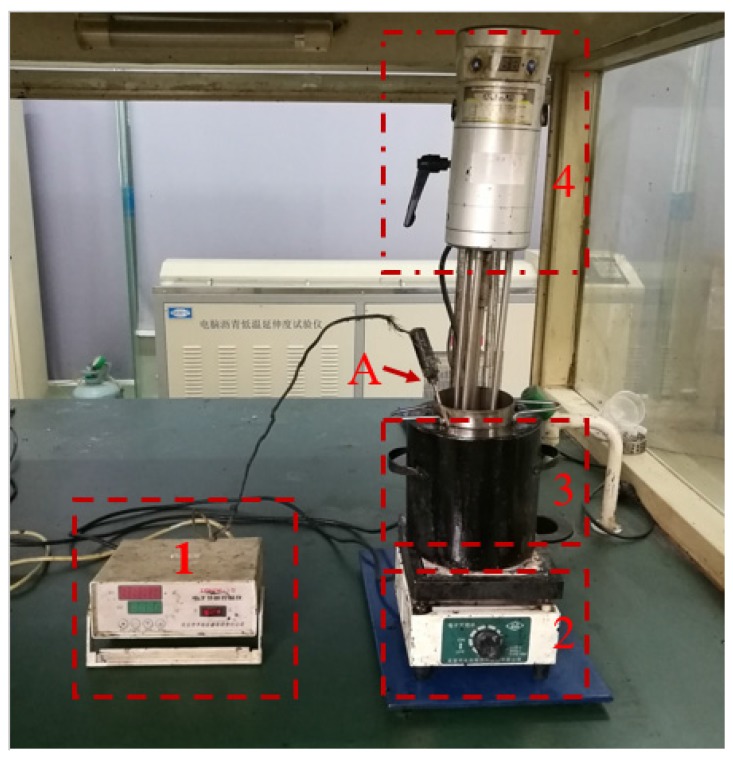
High-speed shearing and intelligent temperature control device. Part 1: Electronic temperature controller: Controls the temperature of bitumen intelligently by connecting with the temperature probe and the electrical resistance furnace. Part 2: Electrical resistance furnace: Heats bitumen intelligently by connecting with the electronic temperature controller. Part 3: Oil bath: Heats bitumen uniformly. Part 4: High speed shearing machine: Provides high-speed shearing and shearing rate control. Part A: Temperature probe: Detects the temperature of bitumen.

(1) 3.5% SBS (determined by weight) was added to 90A virgin asphalt which was at a temperature of 160 °C. The mixture was stirred using a high-speed shearing mixer at 5000 rpm for one hour.
(2) The temperature of the SBS modified bitumen was maintained at 160 °C by heating using an oil bath.
(3) MWCNTs were steadily added into the bitumen for 30 min with a high-speed shearing mixer running at 5000 rpm.
(4) Finally, the high-speed shearing mixer at 5000 rpm was applied for 30 min to disaggregate and disperse the agglomerated MWCNTs into the bitumen matrix. High-speed shearing and intelligent temperature control device was showed in Figure 2. In addition, the normal properties of 90A bitumen and SBS modified bitumen were showed in Table 2.

### 2.3. Instrument and Performance Tests

#### 2.3.1. Conventional Bitumen Tests

The three conventional bitumen indexes were conducted to make comparisons between the SBS modified bitumen and the MWCNTs/SBS modified bitumen. A penetration tester (SYD-2801F, Shangyi, Shanghai, China), softening point tester (SYD-2806F, Shangyi, Shanghai, China), and ductility tester (LYY-7D, ZGHTKY, Cangzhou, China) were used in this paper. They were used to perform the penetration test (0.1 mm at 25 °C, 100 g, 5 s), softening point test, and ductility test (5 cm/min, 5 °C), which were done in accordance with JTG E20-2011 [16].

#### 2.3.2. Brookfield Viscosity

The Brookfield rotational viscometer (THERMOSEL, BROOKFIELD, Stoughton, MA, USA) test was conducted to determine the viscosity temperature susceptibility of the SBS modified bitumen and MWCNTs/SBS modified bitumen at four different temperatures of 120 °C, 135 °C, 150 °C and 165 °C, in accordance with JTG E20-2011 [16].

#### 2.3.3. Dynamic Shear Rheometer (DSR) Tests

A dynamic shear rheometer (DSR) of model MCR101 and manufactured by Anton Paar (Vienna, Austria) was used. A temperature sweep test was conducted using the DSR under strain-controlled mode with a constant frequency of 10 rad/s according to specifications [17]. A temperature sweep from 30 to 80 °C with a temperature increment of 2 °C per minute was designed to investigate the high temperature property of MWCNTs/SBS modified bitumen. Plates with 25 mm diameter and 1 mm gap were used. The performance indicators recorded by DSR were the complex shear modulus (G*) and phase angle (*δ*).

#### 2.3.4. Bending Beam Rheometer (BBR) Tests

In order to obtain the different low temperature performances between the SBS modified bitumen and SBS/MWCNTs, the BBR (TE-BBR, Cannon, New York, NY, USA) test was used to measure the stiffness and m value in accordance to specifications [16]. According to the standard [18], SBS modified bitumen was tested for several temperatures and finally −16 °C was determined as the critical temperature to evaluate the low temperature property of MWCNTs/SBS modified bitumen.

#### 2.3.5. Modification Mechanism Characterization

In order to compare the modification difference between the SBS modified bitumen and MWCNTs/SBS modified bitumen, bitumen’s four components test was conducted in accordance with specifications [16]. In addition, thermal analysis and FTIR tests were also conducted to investigate the modification mechanism.

The thermal analysis test with a working temperature range of 100–700 °C was conducted. A TGA/DSC simultaneous thermal analyzer STA449c/3/G manufactured by NETZSCH (Selb, Germany) was used in this paper. The heating rate was controlled at 20 °C/min with a maximum temperature of 700 °C. At the same time, high-purity nitrogen ambient gas was applied at a flow rate of 500 mL/min. During the pyrolysis process, the organic volatile substances of the polymers were decomposed to low molecular weight products. The relationship between the mass of the test sample and the temperature can be obtained from the TGA tests, and the decomposition temperature of different components can also be obtained from the DTG and DSC curves.

The FT-IR test was conducted to investigate the modification mechanism with wavelengths ranging from 400 to 4000 cm^−1^, using an infrared spectrum instrument Nexus manufactured by Thermo Nicolet Corporation (MA, USA). The FT-IR test can detect functional groups in an organism which can be used to determine whether a chemical reaction occurs by comparing the differences in functional groups.

The concentration change of different components (saturates, aromatics, resins, and asphaltenes) in bitumen was studied by the thin layer chromatography detection (TLC-FID) method. The four components analyzer Iatroscan MK-6 manufactured by IATRON (Japan) was used. Bitumen was dissolved in dichloromethane solution, and then *N*-heptane, toluene/heptane (80:20, *v*/*v*), toluene/ethanol (55:45, *v*/*v*) were used as the first, second, and third extension solvent, respectively, to isolate saturates, aromatics, and resins successively. Organic ions are generated by the high temperature of the hydrogen flame, and FID can detect the current intensity generated by the organic ions. The larger the current intensity, the more content of bitumen components corresponds to this area on the chromatography.

The fluorescence microscopy test was used to detect fluorescence materials in the binder such as SBS. The specimens included the SBS modified bitumen, 1.0% MWCNTs/SBS modified bitumen, and those obtained by the segregation experiment. The segregation test, in accordance with specifications [16], was conducted by heating tubes filled with binder for 48 h at 160 °C to analyze the difference between the upper 1/3 part of the bitumen and the bottom 1/3 part of the bitumen.

For all the tests listed above, three replicates were performed for the different contents of MWCNTs for the same testing conditions.

#### 2.3.6. Experimental Program Outline

**Figure 3:**
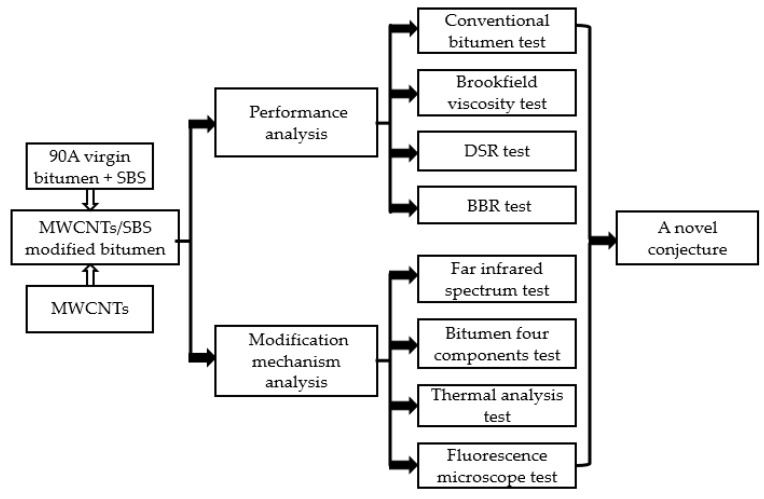
Experimental program outline.

The experimental program outline is shown in Figure 3. Firstly, 90A virgin bitumen, SBS, and MWCNTs were mixed to produce MWCNTs/SBS modified bitumen by high-speed shearing. Secondly, the performance tests and modification mechanism tests were conducted. Finally, a novel conjecture was proposed based on the performance and modification mechanism analysis.

## 3. Results and Discussion

### 3.1. Performance Analysis

#### 3.1.1. Conventional Test Analysis

**Figure 4:**
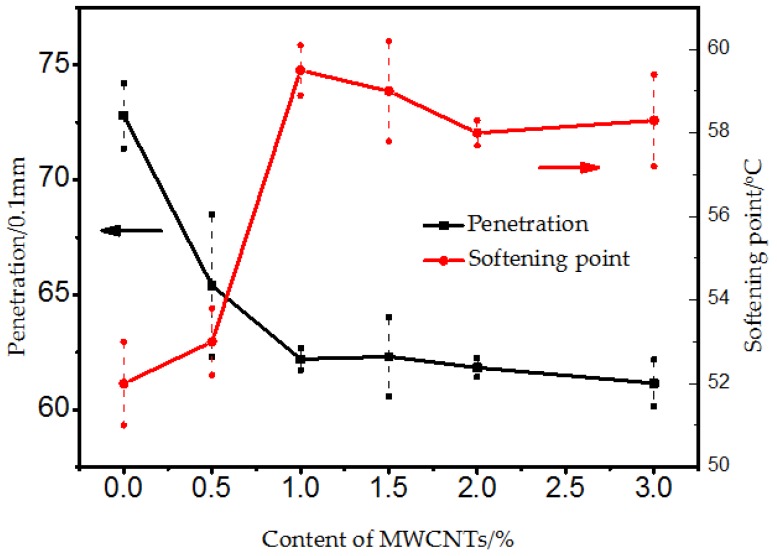
Penetration and Softening point of bitumen with different content of MWCNTs.

Figure 4 shows the effect of the MWCNTs additive on the penetration and softening point of original bitumen. The penetration reflects the softening and hardening degrees of bitumen at moderate conditions. The smaller the penetration value, the harder the bitumen. It was observed that the addition of MWCNTs to the SBS modified bitumen had a significant effect on the penetration resistance. Penetration significantly decreased with the addition of MWCNTs up to 1%. After then, a moderate decrease was observed. This implied that the MWCNTs could significantly improve the hardening degree of the SBS modified bitumen.

The softening point test is commonly used as a standard test for describing an approximate limit between viscous and visco-elastic bitumen behavior, and it reflects the deformation resistance degree of bitumen at high temperature [19]. When the softening point value is higher, the modified bitumen is considered to have a stronger elastic characteristic against bitumen flow. Seen in Figure 4, the softening point had a moderate increase with the addition of 0.5% MWCNTs and a sharp increase with the addition of 1.0% MWCNTs. When more than 1.0% of MWCNTs were added, the softening point value fluctuated and no significant change was observed. It was generally concluded that MWCNTs made the SBS modified bitumen more stable against flowing when subjected to high temperatures, which meant that the MWCNTs/SBS modified bitumen had a better high temperature rutting resistance.

**Figure 5:**
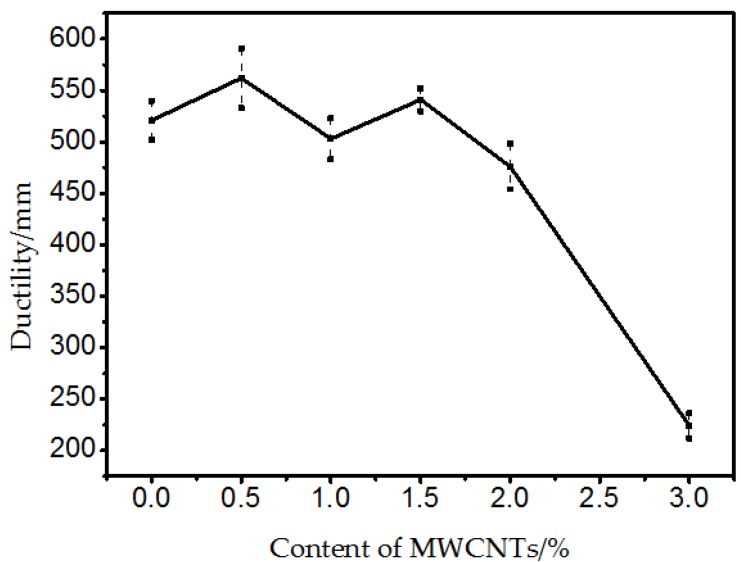
Ductility of bitumen with different content of MWCNTs.

Figure 5 shows the results of the ductility test. There was a small amplitude increase or decrease compared to the original bitumen at 0.5%, 1.0%, 1.5% and 2.0% MWCNTs. A general fluctuation of ductility was observed for MWCNTs addition up to 2.0%. With the addition of 3.0% MWCNTs, there was a significant decrease in ductility. One possible reason was that when the content of MWCNTs was less than 2.0%, there was a complex interaction between the MWCNTs and the SBS modified bitumen which reflected no severe regularity against ductility. However with the addition of 3.0% MWCNTs, the agglomeration of MWCNTs became a stress concentration area which accelerated the fracture process when subjected against tensile stress at low temperature.

#### 3.1.2. Brookfield Viscosity Test Analysis

**Figure 6:**
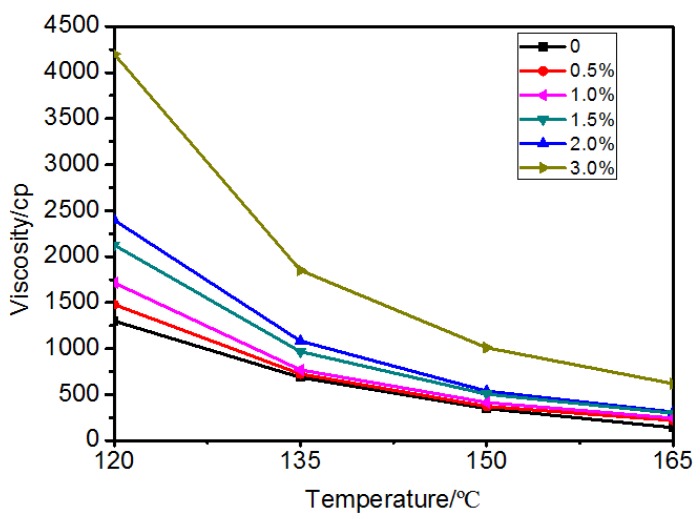
Viscosity versus temperature of bitumen with different content of MWCNTs.

Figure 6 shows the results of the Brookfield viscosity test. The Brookfield viscosity test reflects the frictional resistance which comes from the relative motion between two fluid layers in the bitumen. The higher the viscosity, the better the frictional resistance of bitumen against flowing, and this reflects a better high temperature rutting resistance property. The VTS (Viscosity temperature susceptibility) value was used to evaluate the temperature susceptibility of bitumen. The higher the VTS, the more susceptible the bitumen is to changes in viscosity when subjected to high temperature [20]. From the testing results (Figure 6), it was noted that with the addition of MWCNTs, the viscosity value has a stable increase from 0% to 2%, but with each subsequent addition of higher percentages of MWCNTs, a viscosity increase was observed which was the highest at 120 °C. At 135 °C, the viscosities recorded were all within an acceptable range of Superpave specifications recommended for mixing [21]. When 3.0% MWCNTs was added, the viscosity value had a significant increase.

**Figure 7:**
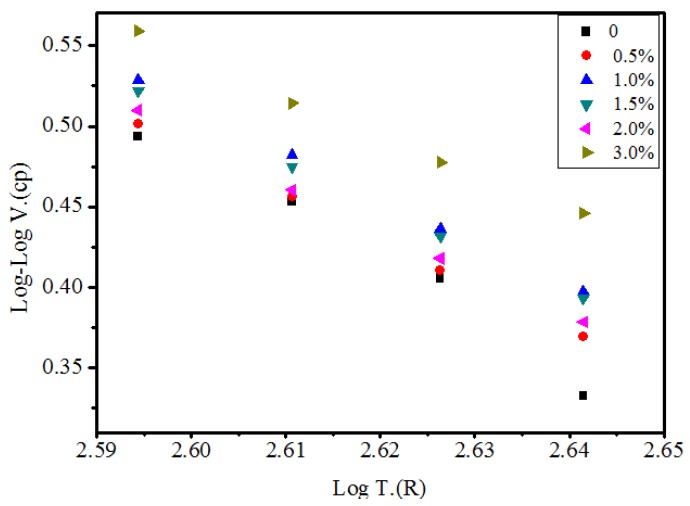
Log-Log V. (cp) versus Log T. (R) with different content of MWCNTs.

Figure 7 shows the Log-Log Viscosity (cp) vs. Log Temperature (R) with different contents of MWCNTs. Table 3 shows the VTS values of the original and modified bitumen along with the coefficient of determination (R^2^). It can be seen that the VTS values decreased with the increase of MWCNTs, indicating lower temperature susceptibility of the bitumen. In view of this, MWCNTs as an additive could improve the high temperature rutting resistance of SBS modified bitumen and decrease temperature susceptibility.

#### 3.1.3. DSR Analysis

**Figure 8:**
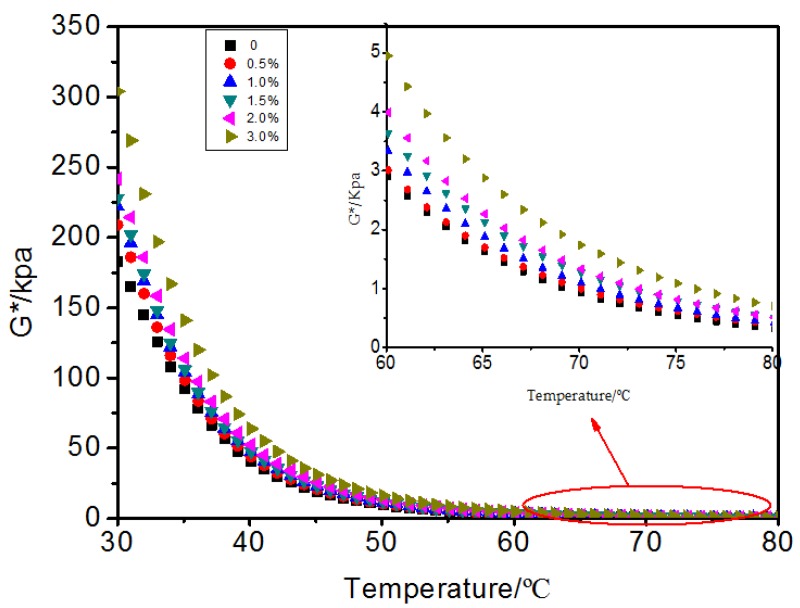
G* versus temperature of bitumen with different content of MWCNTs.

**Figure 9:**
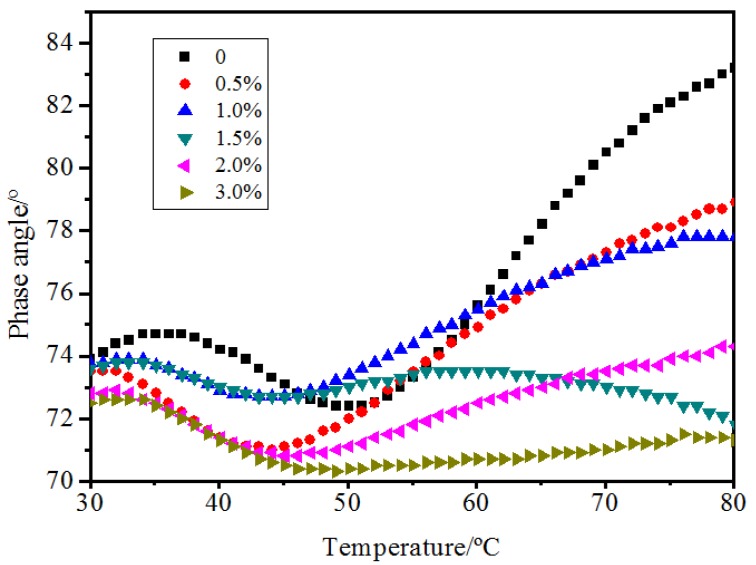
Phase angle versus temperature curve of bitumen with different content of MWCNTs.

**Figure 10:**
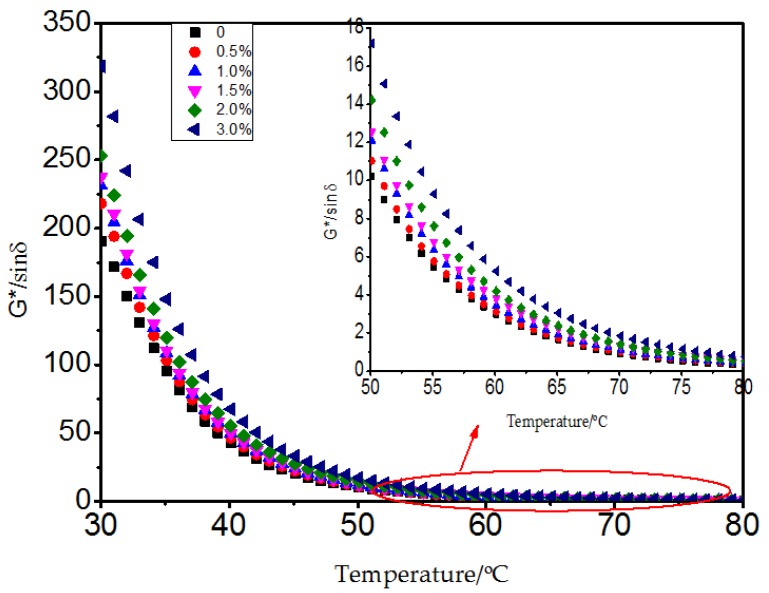
$\;G^{*}/\sin\delta$ versus temperature of bitumen with different content of MWCNTs.

Figure 8, Figure 9 and Figure 10 show the result of the dynamic shear rheometer test. The complex shear modulus describes the stiffness degree of the bitumen, and the higher the modulus, the stronger the property to resist deformation, namely the better rutting resistance of bitumen pavement at high temperature. From Figure 8, the complex shear modulus had a slight increase with the addition of 0% to 2.0% MWCNTs. When 3.0% MWCNTs was added, a significant increase of 25% on average was observed within the temperature range from 30 to 50 °C. The phase angle reflects the proportion between the elastic component and viscous components. The smaller the phase angle, the higher the elasticity recovery when the bitumen pavements are subjected to traffic at high temperatures [22,23]. With the addition of MWCNTs (Figure 9), there was a decrease from 40 to 50 °C which was attributed to the formation of a noticeable elastic network at this temperature by the SBS. When the temperature was higher than 50 °C, the network was destroyed and the phase angle increased with higher temperature. When the temperature was higher than 50 °C, with the addition of MWCNTs, the phase angle had a decreasing trend in general which meant that the content of the viscous components decreased and the content of the elastic components increased. The binder containing 1.0% and 1.5% MWCNTs did not have obvious regularity, which may be attributed to the complex interaction between the bitumen, SBS, and MWCNTs. When adding 3.0% MWCNTs, the variation range of the phase angle with the rise of temperature is far less than for the original SBS modified asphalt. It could be seen that MWCNTs as an additive improved the deformation recovery ability of bitumen. $\;G^{*}/\sin\delta$ versus temperature of the bitumen with different content of MWCNTs was calculated and is shown in Figure 10. It can be seen that the rutting factor increased to a different extent with the addition of MWCNTs which indicated that MWCNTs as an additive improved the high temperature rutting resistance of the SBS modified bitumen. Overall, the DSR test implies that the MWCNTs improved the high temperature performance of the SBS modified bitumen.

#### 3.1.4. BBR Analysis

**Figure 11:**
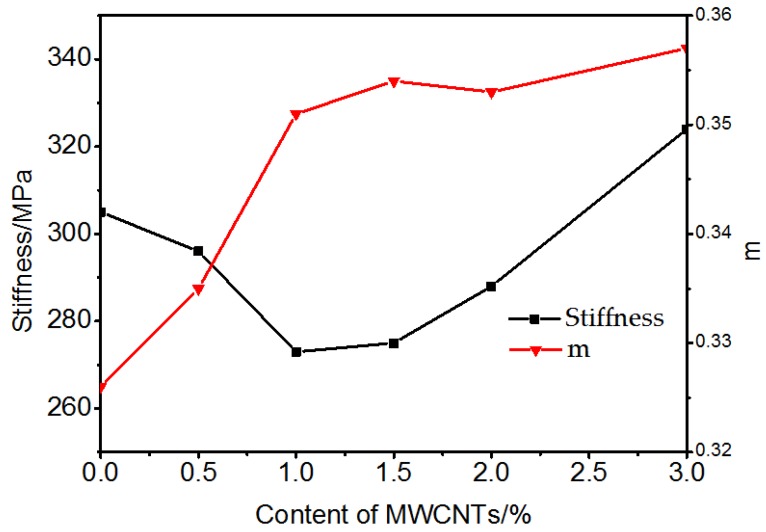
Creep stiffness and m from 60 s at −16 °C.

In order to evaluate the low temperature crack resistance performance of the bitumen, bending beam rheometer (BBR) tests were conducted. SHRP showed that the BBR test had a better correlation with low temperature crack resistance compared to the ductility test. Parameter “m” of the BBR test was related to stress relaxation [24]. As m increased, the stress relaxation property improved. In other words, when subjected to low temperatures, SBS modified bitumen containing MWCNTs is expected to have a better stress relation behavior. S reflected the degree of brittleness. The higher the S value, the lower the cracking resistance at low temperature. According to the standard [17], SBS modified bitumen was tested at several temperatures. When the temperature fell to −16 °C, the stiffness of the SBS modified bitumen was larger than 300 MPa, so finally −16 °C was determined as the critical temperature to evaluate the low temperature property of the MWCNTs/SBS modified bitumen. From Figure 11, it can be seen that with the addition of MWCNTs, the m value generally had an increasing trend, while S slowly decreased with the addition of 0 to 1.5% of MWCNTs. The stiffness was less than 300 MPa when 0.5% to 2.0% MWCNTs was added. When more than 1.5% MWCNTs was added, S had a sharp increase which may result from the agglomeration of MWCNTs, improving the stiffness of the binder. Overall, the addition of MWCNTs improved the low temperature crack resistance of SBS modified bitumen at concentrations less than 2.0%. In addition, it also reflected that the ductility was not the most appropriate evaluation tool of the low temperature performance of bitumen, especially for evaluating MWCNTs/SBS modified bitumen.

### 3.2. Modification Mechanism

#### 3.2.1. Far Infrared Spectrum Analysis

**Figure 12:**
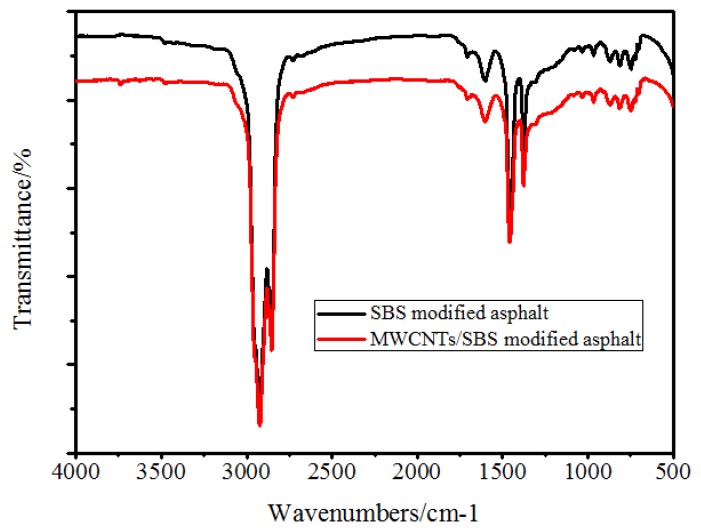
FT-IR of original and 1.0% MWCNTs modified bitumen.

From the FT-IR test (Figure 12), no new chemical functional groups were formed with the addition of MWCNTs, and all the different concentrations of MWCNTs showed a similar trend. This meant that the MWCNTs did not chemically react with bitumen, or that the interaction was so weak that the FT-IR test could not detect it directly.

#### 3.2.2. Bitumen Four Components Analysis

Table 4 shows the bitumen four components test. It was seen that with the addition of MWCNTs, the contents of saturates and aromatics decreased. On the other hand, the contents of resins and asphaltenes increased. One possible reason for this is that the MWCNTs have a strong adsorption capacity with organic molecules containing benzene rings, such as the aromatics and polystyrene of SBS, through π–π intermolecular forces. In such a case, SBS would have better compatibility with bitumen which meant that more saturates were absorbed in the network of SBS and then a stronger structure was formed. Saturates which filtered into the network of SBS could not be washed out by the solvent used in the bitumen four components test [25]. As a result, the percentage of light components decreased and the percentage of heavy portions such as resins and asphaltenes increased, respectively, which gave an increased viscosity and softening point of the bitumen. The result was in agreement with the Brookfield viscosity and softening point test. At concentrations higher than 2.0%, the agglomeration of MWCNTs played a major role in the bitumen and agglomerated MWCNTs lose this specific property and thus the percentage of the bitumen four components did not change.

#### 3.2.3. Thermal Analysis

**Figure 13:**
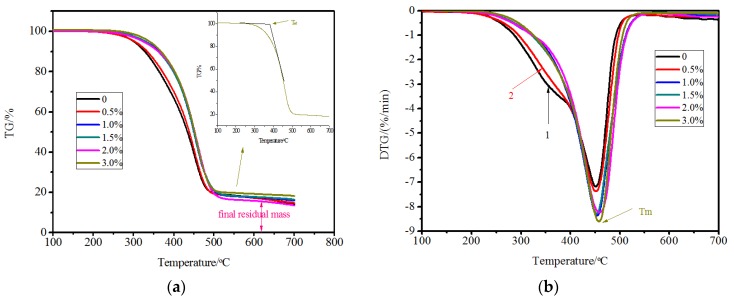
Thermal analysis of bitumen with different content of MWCNTs: (**a**) TG versus temperature; (**b**) DTG versus temperature.

The thermal analysis test was conducted to investigate the thermal decomposition pattern of the test samples. Thermal Gravimetric analysis (TG) and Derivative Thermogravimetry analysis (DTG) were performed to analyze the thermal stability and decomposition of different phases. Parameters T_ed_, T_m_, and M_f_ were used to evaluate the thermal stability of the materials, in which T_ed_ is the epitaxial decomposing temperature, T_m_ is the temperature corresponding to the maximum mass loss rate, and M_f_ is the final residue mass ratio [26,27,28,29]. The DTG curve was used to distinguish the different components in bitumen. Figure 13a shows that the SBS modified bitumen with 0% and 0.5% of MWCNTs decomposed at an earlier time. An improvement was observed at a MWCNTs concentration of 1.0%, and after then, only a slight improvement was observed.

Compared to the DTG curves, it was observed that there was a slight step before T_m_ namely in area 1 (the curve part obviously lower than other content MWCNTs/SBS modified bitumen between 300 °C and 400 °C) in Figure 13b. The DTG from 300 to 400 °C was mainly attributed to the decomposition and volatilization of saturates and aromatics [30]. A similar step also exists in Figure 13b, namely 2 (the curve part slightly higher than area 1 between 300 °C and 400 °C), but was slightly higher which indicated that the decomposition rate of the light components decreased. At 1.0% concentration, the DTG curve became smooth and did not show the steps.

Table 5 shows the parameters T_ed_, T_m_, and M_f_ for the six different concentrations of MWCNTs. It was generally observed that with the addition of MWCNTs, the T_ed_, T_m_, and M_f_ increased which meant that the MWCNTs improved the thermal stability of the SBS modified bitumen. This better performance should be attributed to the stronger network structure which prevented the decomposition and volatilization of light components. As a result, the value of DTG regarding the saturates and aromatics decreased. Thermal analysis confirmed the testing results of the bitumen four components test analysis.

#### 3.2.4. Fluorescence Microscopy Test

**Figure 14:**
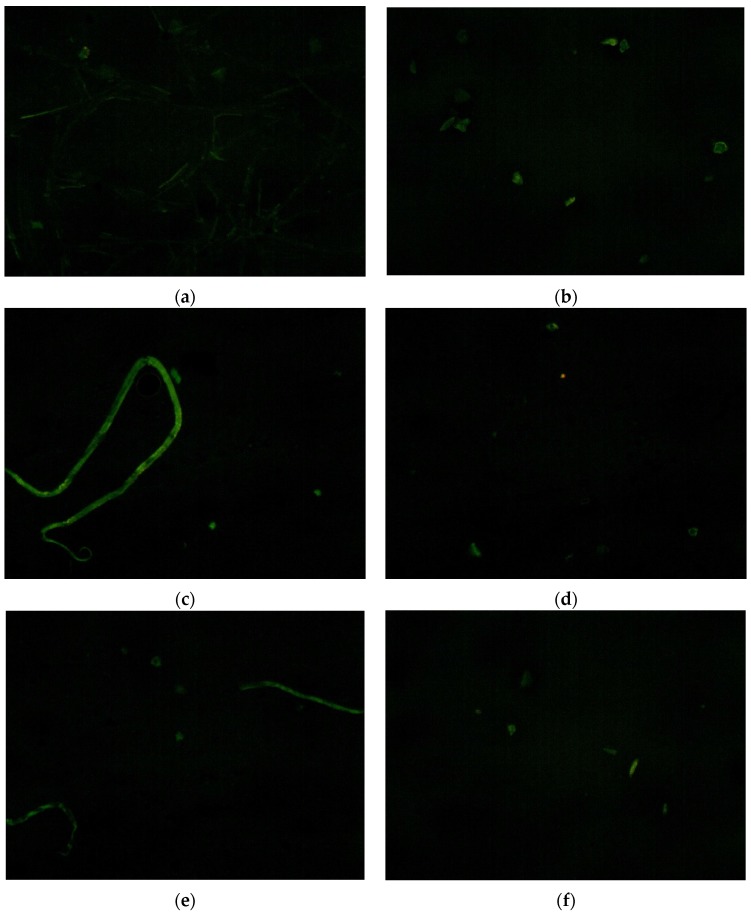
Fluorescence microscopy from ×100: (**a**) SBS modified bitumen; (**b**) 1.0% MWCNTs/SBS modified bitumen; (**c**) the upper 1/3 part of the tube filled with SBS modified bitumen; (**d**) the bottom 1/3 part of the tube filled with SBS modified bitumen; (**e**) the upper 1/3 part of the tube filled with 1.0% MWCNTs/SBS modified bitumen; (**f**) the bottom 1/3 part of the tube filled with 1.0% MWCNTs/SBS modified bitumen.

The fluorescence microscopy test was conducted to analyze the dispersion and storage stability of PmB. SBS produces fluorescence under UV irradiation which can be used to detect the traces of SBS. From the performance tests, we concluded that 1.0% MWCNTs was the optimum content, so SBS modified bitumen and 1.0% MWCNTs/SBS modified bitumen was tested to determine the difference. From Figure 14a,b, the existent morphology of SBS changed from floccule to granule and SBS has a better dispersion in Figure 14b. The flocculent structure is one stage of SBS agglomeration. Figure 14c shows that a large number of SBS grains agglomerated and became clear ribbons and there were more slight and vague ribbons in Figure 14e. In addition, the content of SBS in Figure 14d is less than that in Figure 14f. Overall, Figure 14a,b shows that the MWCNTs improved the dispersion of SBS in the bitumen, and Figure 14e–f indicate that the MWCNTs improved the storage stability of the SBS modified bitumen. There may be a complex interaction between the bitumen, SBS, and MWCNTs which resisted the agglomeration of SBS and promoted the dispersion and dissolution of the SBS in bitumen.

**Figure 15:**
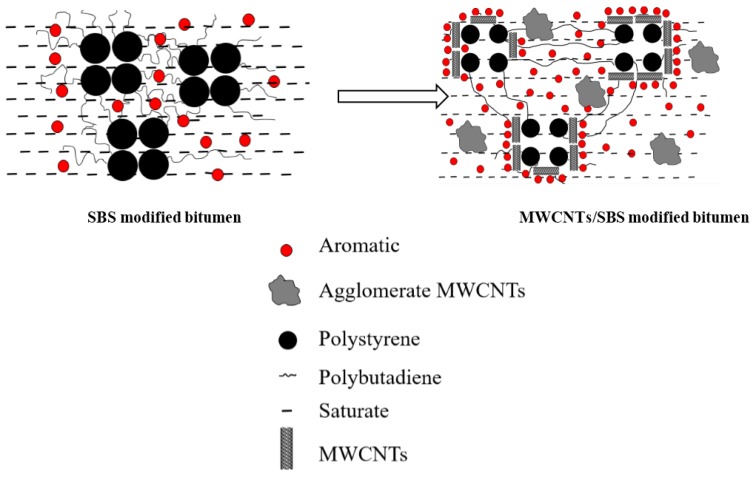
Schematic of MWCNTs/SBS modified bitumen.

A strong π–π interaction between the aromatic molecules and MWCNTs was noted from the study, and the adsorption of carbon nanotubes on the saturated molecules was much less than that of the organic molecules containing benzene rings [31,32,33]. In view of this, a conjecture about the specific interaction between MWCNTs and SBS modified bitumen was proposed to explain why the MWCNTs improved the performance of the SBS modified bitumen: One end of the MWCNTs could have formed a non-covalent complex with the polystyrene of the SBS through π–π conjugates, and the other part could also have a π–π conjugate action with aromatics. This implied that the MWCNTs could make SBS have a better compatibility with bitumen and then more saturates and aromatics would be filtered into the SBS network structure. As a result, a stronger network structure consisting of SBS, light components of bitumen, and MWCNTs was formed which yielded the better high temperature rutting resistance and low temperature crack resistance of bitumen. This explanation is in agreement with the performance tests and the mechanism and structure analysis. According to the above explanation, the schematic in Figure 15 was proposed.

## 4. Conclusions

In this paper, experiments were conducted according to two different approaches: one was to investigate the effect of adding MWCNTs on the properties of SBS modified bitumen at high and low temperatures, and the other was to explain the modification mechanism according to the specific structure and properties of MWCNTs, as well their the interaction with SBS bitumen. Five different percentages (0.5%, 1.0%, 1.5%, 2.0% and 3.0%) of MWCNTs were blended with SBS modified bitumen. the following conclusions can be drawn:

1. MWCNTs as an additive had a positive effect on the performances of the SBS modified bitumen. The optimum concentration of MWCNTs in SBS modified bitumen was determined as 1.0%.
2. The Brookfield rotational viscosity test showed that the MWCNTs as an additive improved the high temperature susceptibility of the SBS bitumen. The DSR test showed that MWCNTs could improve the high temperature property of the SBS modified bitumen and the BBR test indicated that MWCNTs improved the low temperature crack resistance.
3. The IR test revealed that there is no new chemical functional groups formed by the addition of MWCNTs. The bitumen four components test showed that MWCNTs as an additive to SBS modified bitumen result in changes in the content of each component. The thermal analysis test confirmed that the MWCNTs improved the thermal stability of the SBS modified bitumen and the light components decomposed at a slower rate. In addition, the fluorescence microscopy test showed that the MWCNTs improved the dispersion and storage stability of SBS in bitumen. A schematic was proposed by the strong adsorption property due to π–π interaction between MWCNTs and organic molecules that containing benzene rings, to explain why MWCNTs had a positive effect on the SBS modified bitumen. It was suggested that MWCNTs act like a ‘bridge’; one of its ends had a π–π conjugated complex with the polystyrene of SBS, and the other side had a π–π conjugate interaction with the aromatic molecules. There was a better compatibility of polystyrene and for that reason, more saturates and aromatics filtered into the network structure of SBS and then a stronger network structure consisting of SBS, light components of bitumen, and MWCNTs in the MWCNT/SBS modified bitumen was formed. The improved high and low temperature performance was attributed to the stronger network structure. Further study is needed to directly confirm the existence of π–π interactions between the polystyrene of SBS and the MWCNTs in bitumen.

These conclusions are only limited to the materials used in this study and they may be different for other materials.

## Figures and Tables

**Table 1:** Physical properties of multi-wall carbon nanotubes (MWCNTs) supplied by the manufacturer.

Purity	Internal Diameter	Outside Diameter	Length	Surface Area	Density	Preparation Method
>95/% (by weight)	3–5 nm	8–10 nm	3–12 μm	>200 m^2^/g	0.1 g/cm^3^	Chemical vapor deposition

**Table 2:** Properties of virgin binder and SBS modified asphalt.

Physical Properties	90A	SBS Modified Bitumen
Penetration (25 °C, 0.1 mm)	84.7	72.8
Softening point (°C)	47.8	52.0
Ductility (5 cm/min, 5 °C, 1 mm)	/	521
Viscosity (135 °C, pa·s)	0.47	0.69

90A is an abbreviation of bitumen with 80/100 pen grade. The content of MWCNTs (namely x%) involved in this paper was mass percentage relative to the binder.

**Table 3:** Viscosity temperature susceptibility (VTS) of MWCNTs/SBS modified bitumen.

Content of MWCNTs/%	0	0.5	1.0	1.5	2.0	3.0
VTS	−3.3642	−2.8098	−2.8135	−2.7363	−2.783	−2.4001
R^2^	0.9765	0.9997	0.9994	0.9992	0.9987	0.9961

**Table 4:** Proportion of MWCNTs/SBS bitumen four components.

The Percentage of MWCNTs/%	The Content of Components/%			
	Saturation	Aromatic	Resin	Asphaltene
0	17.27	45.22	32.65	4.86
0.5	15.99	44.52	34.12	5.37
1.0	14.50	42.19	37.85	5.46
1.5	14.11	42.18	38.08	5.63
2.0	14.04	42.29	38.17	5.50
3.0	14.32	42.01	37.74	5.93

**Table 5:** The T_ed_ (epitaxial decomposition temperature), the T_m_ (temperature of the fastest decomposition rate), and the M_f_ (final residual mass) of MWCNTs/SBS modified bitumen.

The Percentage of MWCNTs/%	T_ed_/°C	T_m_/°C	M_f_/%
0	364.5	451.4	14.23
0.5	374.1	451.4	14.78
1.0	390.9	453.7	15.92
1.5	393.9	453.3	16.52
2.0	397.9	459.2	13.61
3.0	397.1	457.6	18.30
